# Agro-Environmental Determinants of Avian Influenza Circulation: A Multisite Study in Thailand, Vietnam and Madagascar

**DOI:** 10.1371/journal.pone.0101958

**Published:** 2014-07-16

**Authors:** Mathilde C. Paul, Marius Gilbert, Stéphanie Desvaux, Harena Rasamoelina Andriamanivo, Marisa Peyre, Nguyen Viet Khong, Weerapong Thanapongtharm, Véronique Chevalier

**Affiliations:** 1 Centre de coopération internationale en recherche agronomique pour le développement (CIRAD), UR AGIRs, Montpellier, France; 2 Université de Toulouse, INP-ENVT, UMR ENVT INRA 1225 IHAP, Toulouse, France; 3 Biological Control and Spatial Ecology, Université Libre de Bruxelles, Brussels, Belgium; 4 Fonds National de la Recherche Scientifique, Brussels, Belgium; 5 Direction Régionale de l’Alimentation, de l’Agriculture et de la Forêt de Languedoc- Roussillon, Montpellier, France; 6 FOFIFA-DRZV, Antananarivo, Madagascar; 7 Faculté de Médecine d’Antananarivo - Département Vétérinaire, Antananarivo, Madagascar; 8 National Institute of Veterinary Research, Hanoi, Vietnam; 9 Department of Livestock Development, Bangkok, Thailand; Harvard School of Public Health, United States of America

## Abstract

Outbreaks of highly pathogenic avian influenza have occurred and have been studied in a variety of ecological systems. However, differences in the spatial resolution, geographical extent, units of analysis and risk factors examined in these studies prevent their quantitative comparison. This study aimed to develop a high-resolution, comparative study of a common set of agro-environmental determinants of avian influenza viruses (AIV) in domestic poultry in four different environments: (1) lower-Northern Thailand, where H5N1 circulated in 2004–2005, (2) the Red River Delta in Vietnam, where H5N1 is circulating widely, (3) the Vietnam highlands, where sporadic H5N1 outbreaks have occurred, and (4) the Lake Alaotra region in Madagascar, which features remarkable similarities with Asian agro-ecosystems and where low pathogenic avian influenza viruses have been found. We analyzed H5N1 outbreak data in Thailand in parallel with serological data collected on the H5 subtype in Vietnam and on low pathogenic AIV in Madagascar. Several agro-environmental covariates were examined: poultry densities, landscape dominated by rice cultivation, proximity to a water body or major road, and human population density. Relationships between covariates and AIV circulation were explored using spatial generalized linear models. We found that AIV prevalence was negatively associated with distance to the closest water body in the Red River Delta, Vietnam highlands and Madagascar. We also found a positive association between AIV and duck density in the Vietnam highlands and Thailand, and with rice landscapes in Thailand and Madagascar. Our findings confirm the important role of wetlands-rice-ducks ecosystems in the epidemiology of AI in diverse settings. Variables influencing circulation of the H5 subtype in Southeast Asia played a similar role for low pathogenic AIV in Madagascar, indicating that this area may be at risk if a highly virulent strain is introduced.

## Introduction

A new, atypical influenza virus infection caused by the H7N9 subtype [1] emerged in March 2013 in Eastern China. As of 25 October 2013, it has been associated with 137 confirmed human infections and 45 related deaths [2]. This event raises renewed questions regarding the potential of avian influenza viruses to infect humans and of human pandemicity [3]. Controlling the spread of avian influenza viruses (AIV) in poultry may contribute to reducing the risk for human infection by limiting poultry-to-human transmission and preventing the emergence of a viral form with efficient human-to-human transmission [4]. To achieve this goal, a better understanding of the local conditions that favor the circulation of avian influenza viruses in poultry is necessary.

The degree to which AIV spread and are maintained in poultry populations is highly variable and may partly be explained by variations in landscape or agro-environmental features around farms [5]. Three main drivers for the spatial distribution of Highly Pathogenic Avian Influenza (HPAI) H5N1 outbreaks have been identified in previous studies [6]. The first is density of domestic ducks, which can shed the virus with minimal clinical signs and spread it silently in poultry populations [7]. The second is anthropogenic variables, as the risk of HPAI H5N1 has been found to be greater in areas that have high human population densities and are located close to transportation networks [8], [9]. This may be explained by a higher probability of outbreak detection in these areas, but also by an increased virus transmission through movements of contaminated poultry or fomites. The third driver consists of water-related variables [10]–[13] because AIV can persist in water for extended periods of time [14].

Many studies have examined associations between agro-environmental factors and the presence of avian influenza outbreaks [5], [8], [9], [11]–[13], [15]–[25]. The comparison, based on field data, of risk factors across countries would be useful for analyzing similarities and differences in transmission patterns and subsequently improving detection and control policies. However, such a comparison is complicated by several factors. First, the studies involved used different data collection protocols and analytical methods, rendering comparisons across countries difficult. Second, while correlations between agro-environmental determinants and AIV persistence have been fairly well-studied at a broad scale, the processes operating at a fine geographic scale are complex and remain poorly documented [6]. Third, although the geographic co-distribution of H7N9 outbreaks with H5N1 outbreaks in China suggests that some areas might be a common ground for transmission of emerging low and highly pathogenic AIV [26], the current lack of studies on low pathogenic AIV renders it difficult to draw conclusions.

In our multisite study, we aimed to identify and compare the local combinations of environmental factors associated with avian influenza circulation in different settings. In parallel, we applied the same analytic framework to study fine-scale data collected from four contrasting study sites. One was in Thailand, where HPAI viruses have circulated in the past, two were in Vietnam, where HPAI viruses have and continue to circulate, and the last was in Madagascar, where HPAI viruses have not yet been detected. Despite remarkably similar features with Asian agro-ecosystems, only low pathogenic avian influenza circulation has been found in Madagascar to date [27]. Findings from this study should help to identify local combinations of risk factors for AIV circulation, and thus could be useful for tailoring prevention, surveillance and control at a very fine spatial scale.

## Data and Methods

### General characteristics of the study sites

The present work is part of a large-scale research project, “GRIPAVI”, launched in 2007 to improve understanding of the ecological and epidemiological factors involved in the maintenance and spread of avian influenza viruses in tropical countries. In collaboration with national and international research groups, field surveys were conducted over five years in six study sites located in Vietnam, Mauritania, Mali, Ethiopia, Madagascar and Zimbabwe [28]. To develop a comparative approach, we selected two GRIPAVI sites with similar agro-ecosystems based on rice cultivation and duck farming: the Red River Delta in Vietnam and Lake Alaotra in Madagascar. We added two other study sites featuring similar ecological characteristics, one in the Vietnam highlands, and the other in lower-Northern Thailand, where we previously had conducted extensive field work to gather information on AIV [22], [23].

The study thus covered a total of four sites in three countries (Thailand, Vietnam and Madagascar). These sites all featured agro-ecosystems with rice cultivation and duck farming, but varied in terms of human population density, poultry density, farming techniques (extensive or intensive) and AIV situations.

**Figure 1:**
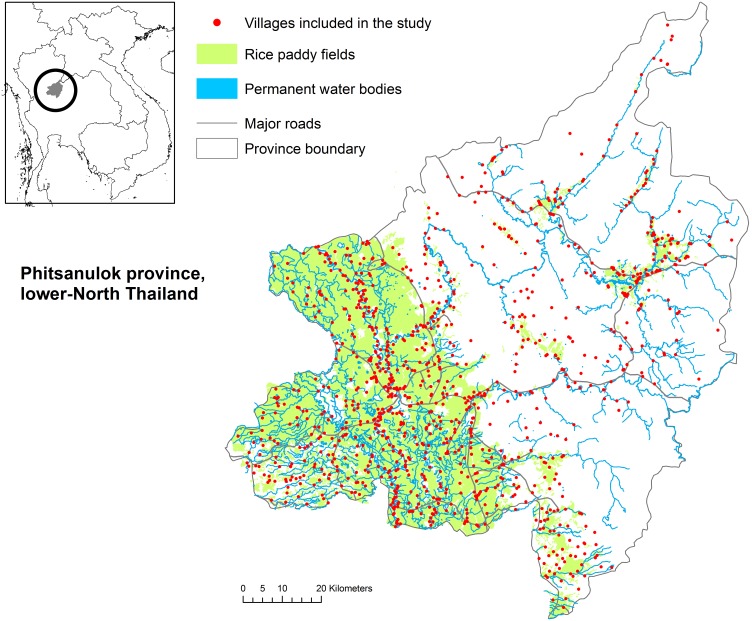
Study area in lower-Northern Thailand. Phitsanulok province and location of the 1032 villages included in the study.

In Thailand, the study area was the province of Phitsanulok (Figure 1), which recorded the highest number of HPAI H5N1 outbreaks in chickens during the 2004–2005 epidemics. This province is located in the Yom-Nan River basin in the lower-Northern region, where the H5N1 virus re-emerged in 2008 and where there is a strong need for studies on the conditions of HPAI outbreak occurrence [29]. Landscapes of this province include plain areas characterized by intensive rice cultivation, with multiple rice crops per year, and large flocks of free-grazing ducks raised on rice fields. The eastern part of Phitsanulok province also covers sparsely populated, higher altitude areas with forests and diverse agricultural crops.

**Figure 2:**
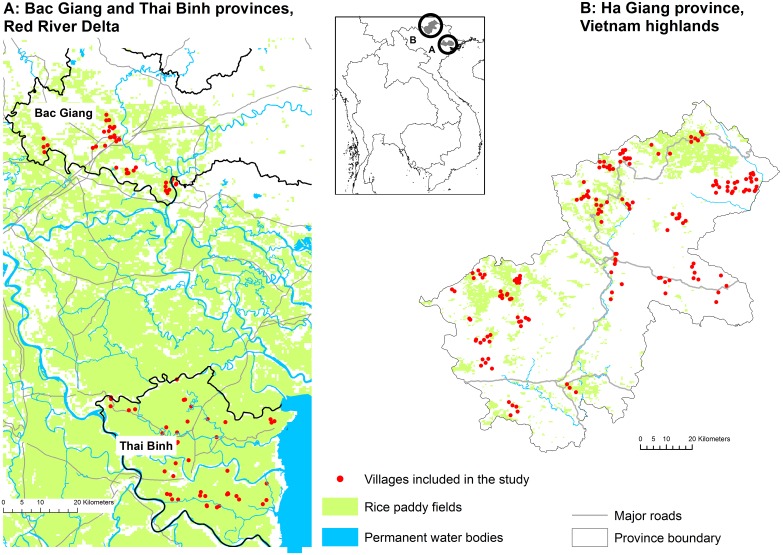
Study area in Vietnam. 2A: Bac Giang and Thai Binh provinces, in the Red River Delta, and location of the 83 villages included in the study. 2B: Ha Giang province, in the Vietnam highlands, and location of the 167 villages included in the study.

In Vietnam, two study sites were selected in two regions presenting contrasting agro-ecological features. The first study site was in the Red River Delta, which is characterized by low elevations and a combination of duck farming with intensive rice cultivation. This site was chosen because HPAI H5N1 outbreaks in Northern Vietnam mainly were concentrated in this type of agro-ecological area [30]. Two provinces were selected: Bac Giang and Thai Binh (Figure 2). The second site was Ha Giang province in the Vietnam highlands (Figure 2), a mountainous area characterized by low human and poultry densities. Rice cultivation is mainly extensive, with only once rice crop cultivated each year. The site was chosen because this part of Vietnam has been studied little yet may be exposed to an increased risk of AIV introduction due to its proximity to the Chinese border [23].

**Figure 3:**
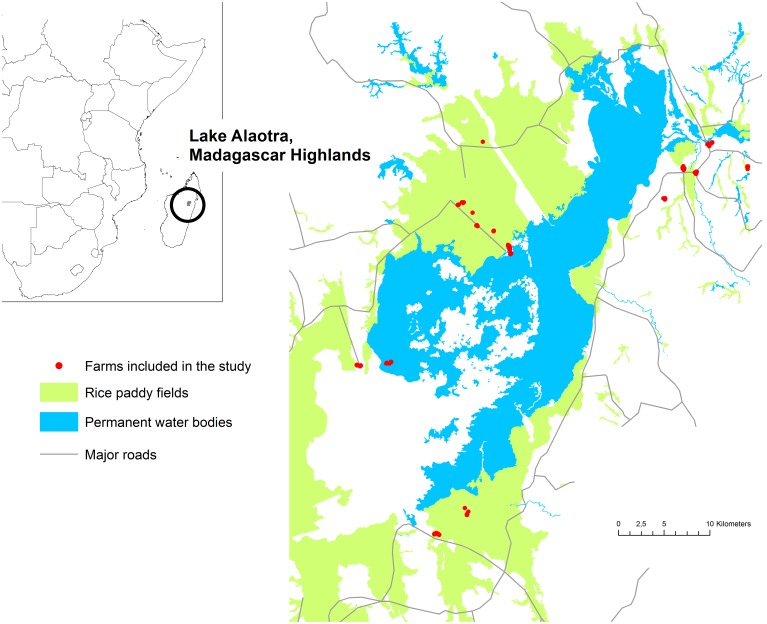
Study area in Madagascar highlands. Lake Alaotra and the 147 farms included in the study.

The fourth study site was the Lake Alaotra region in Madagascar (Figure 3). Madagascar is an Indian Ocean island lying 400 km off the eastern coast of southern Africa. Located 750 m above sea level, the Lake Alaotra region constitutes the largest wetland area in Madagascar, and is a rich habitat for wild birds [31]. It is the largest rice production basin in the country and also is the site of important poultry production activities. There are small-scale chicken farms and ducks and geese are allowed to graze on rice paddies. The following five municipalities located on the lakeside were included in the study: Andromba, Imerimandroso, Ampitatsimo, Anororo, and Amparafaravola.

### Data on avian influenza

The data collected in the four study sites differed due to the distinctive features of each site. H5N1 data was collected in Thailand, H5 seroprevalence data at multiple time points in Vietnam, and cross-sectional low-pathogenic AIV seroprevalence data in Madagascar. Due to differences in the sampling protocols of the preliminary surveys used on the four sites, we had to re-arrange the data and redefine the epidemiological unit. We worked at the finest unit for which spatial coordinates were available. This unit was the village in Thailand and the two sites in Vietnam (Red River Delta and the highlands), and the farm in Madagascar. Outbreaks and prevalence data collected at various dates across study sites (Figure S1) were aggregated over time with the aim to obtain an overall view of the AIV situation for each study site.

In Thailand, data on HPAI H5N1 outbreaks were collected by the Department of Livestock Development (DLD). We restricted the dataset to the second wave of the epidemic, from July 2004 to May 2005, when the surveillance system guaranteed good detection. In addition to routine surveillance and mandatory clinical reporting, two intensive active surveillance programs known as ‘X-ray surveys’ were implemented, with volunteers conducting door-to-door investigations to check poultry nationwide [21]. The virus was confirmed in sick or dead birds and cloacal samples from poultry and wild birds by diagnostic laboratories using reverse-transcriptase polymerase chain reaction and virus isolation [9]. All of the 1032 villages listed in the Phitsanulok administrative database were included in our comparative study.

In Vietnam and Madagascar, data on avian influenza were obtained from previous serological surveys using multistage sampling strategies that have been described elsewhere [23], [27], [32]. Briefly, two repeated cross-sectional surveys were conducted in the Red River Delta (Vietnam). They were based on a stratified, one-stage clustered design with a random selection of the clusters (the flocks for semi-commercial farms or the villages for backyard poultry) and a random selection of the birds within each cluster. Poultry samples were collected in December 2008, January, March and June 2009 (first survey), then monthly from March to June 2011 (second survey). In total, 3234 sera were collected from unvaccinated chickens and ducks in 83 villages and tested using a HI (hemagglutination inhibition) H5 test. In the Vietnam highlands, a serological survey was carried out with poultry samples collected monthly from April 2005 to September 2006. Within each district of Ha Giang province, four communes were chosen to represent all of the ethnic groups and to cover a wide range of environmental conditions and farming systems. Six to eight villages per commune, 6–8 farmers per village, and one or two birds in each household then were randomly selected. Our study finally included 1531 chicken sera collected in 167 villages and tested using an AIV type A ELISA competition test and a H5 pseudoviral particles assay. In Madagascar, the serological survey was based on a stratified, two-stage random sampling scheme with villages and farmers as the first- and second-degree units. A sampling fraction of 10% of villages within municipalities, and 30% of farmers within villages was set as the sampling objective. Our database included 980 sera collected in August 2008 (n = 276) and May 2009 (n = 704) from chickens, ducks and geese on 147 farms located in 14 villages. Sera were tested against avian influenza type A using an ELISA competition test (IDVET©).

### Data on putative spatial risk factors

This study aimed to examine in detail a limited number of risk factors common to all four study sites for which very fine-scale data were available. The variables selected for this study were those which have been found to be significant in various countries according to a recent literature review [6]: poultry (chicken and duck) densities around villages, landscape with predominant rice cultivation, distance to the closest water body and major road, and human population density around villages.

Poultry census data were collected in Thailand at the village level during the X-ray survey organized in February 2005 by the DLD. Chicken and duck densities (birds/km^2^) were calculated using a village area estimated from Delaunay triangulation between village centroids. In Vietnam, chicken and duck densities were estimated by extracting mean values from poultry density rasters [33] in a 1-km radius buffer around village centroids. In Lake Alaotra (Madagascar), poultry density were calculated at the subdistrict level (3rd administrative level), from census data collected in May–June 2008 [27]. Gridded human population datasets were available at a 100-m resolution in all countries for the year 2010 (http://www.afripop.org/; http://www.asiapop.org/). We extracted from these raster layers the mean values of human population density in a 1-km buffer around each study location.

Layers of the road networks were obtained from the Royal Thai Survey Department in Thailand, the National Cartography House in Vietnam, and from DIVA-GIS (http://www.diva-gis.org/) in Madagascar. Euclidian distances from all study locations to the closest major road were calculated. Environmental variables were obtained for the four sites through diverse means. In Thailand, a land cover map including rice fields, rivers, lakes, canals and ponds was provided by the Royal Thai Survey Department. In Vietnam, a series of MODIS images collected in 2005 at 500-m resolution (https://lpdaac.usgs.gov/products/modis_products_table/mod09a1) were processed to map water bodies and rice paddy fields, using methods described elsewhere [34], [35]. In Madagascar, one Landsat 7 enhanced thematic mapper plus (ETM+) satellite image centered on Lake Alaotra, of 30-m resolution and dated from March 2007 was analyzed using supervised classification [36]. Landcover classifications in Vietnam and Madagascar were validated by field visits [16], [36]. The Euclidian distances from each study location (village or farm) to the nearest permanent body of water were calculated. The percentage of area occupied by rice paddy fields in a 1-km radius around points was calculated. Before modeling, distances as well as human and poultry densities were log-transformed to improve the fit of the linear relationship between these variables and the outcome. The rice variable was dichotomized to discriminate landscapes with predominant rice production (percentage of paddy fields in a 1-km neighborhood ≥0.80) from others.

All geoprocessing operations were realized using ArcGIS© software v.9.3 (Esri Inc.) and HawthsTools software v.3.27 (2002–2006 Spatial Ecology LLC).

### Statistical analysis

The statistical analysis was based on spatial generalized linear models, which were run using the MASS package of the R software. Due to differences in data collection, the dependent variable was binomial (village infected/not infected by H5N1) in Thailand, while it was the proportion of seropositive birds in Vietnam (H5 village-level seroprevalence) and Madagascar (farm-level AIV type A seroprevalence). Putative risk factors were first screened using univariate analysis. In a second step, multivariate models were run, including all of the significant covariables from the univariate analysis (*p*-value<0.25, Wald test). Pair-wise correlations between variables and multicollinearity were examined through values of Spearman correlation coefficient (ρ) and variance inflation factors (VIF) [37]. A stepwise backward selection was carried out until all of the remaining variables were significant (*p*≤0.05). As avian influenza is a contagious disease, villages or farms located close to each other may exhibit more similar values of prevalence than those located further apart. This spatial dependency between observations was accounted for by introducing a correlation structure in the univariate and multivariate models (see details in Material S1). The extent of spatial autocorrelation was specified according to the range estimated from the spline correlogram of influenza positivity (Thailand) or prevalence (Vietnam, Madagascar) data. An exponential function was selected for the correlation matrix, as indicated by the shape of the spatial correlogram. To verify whether spatial autocorrelation was correctly accounted for, we inspected the residuals of the logistic models using a Monte Carlo method. This consisted of comparing the observed variogram with variogram ‘envelopes’ that were computed by simulating 999 permutations of the data values across locations [38]. Goodness-of-fit of the models was evaluated by using Hosmer-Lemeshow Chi-squared test.

## Results

In lower-Northern Thailand, 163 out of the 1032 villages (15.8%) in Phitsanulok province had laboratory-confirmed HPAI H5N1 outbreaks between July 2004 and 2005. In the Red River Delta (Vietnam), we found a bird-level H5 seroprevalence of 13.0% in non-vaccinated poultry. Seropositive birds were found in 47 out of the 83 villages (56.6%) included in the study. In the Vietnam highlands, bird-level H5 seroprevalence was 3.3% and 20 villages out of 167 (11.9%) had at least one H5 seropositive bird. In Madagascar, we found a bird-level seroprevalence of 14.6% for low pathogenic AIV. Seropositive birds were found on 74 out of 147 farms (50.3%). The range of spatial autocorrelation estimated from correlograms was 35 km in Thailand, 25 km in the Vietnam highlands, 10 km in the Red River Delta of Vietnam, and 1 km in Madagascar (Figure S2). The variograms computed from the observed residuals of the models lay within the 95% limits of the simulation envelopes (Figure S3). This did not show evidence of unaccounted spatial pattern in the models.

Agro-environmental characteristics contrasted between the four study sites (Table 1). Out of the 6 variables studied, 1 to 6 (that is 1 variable in the Red River Delta, 4 in the Vietnam highlands, and 5 in lower-Northern Thailand and Madagascar, respectively) were found significantly (*p*<0.25) associated with AIV in the univariate analysis (Table 2). The simultaneous introduction of chicken and duck density in the models resulted in a multicollinearity problem for the Vietnam highlands and Madagascar (VIF>5), which may partly be due to significant correlation between these two variables (ρ>0.6). Given its previously established role in the epidemiology of avian influenza [7], only duck density was finally included in the multivariate models. After backward selection, the final model included one variable in the Red River Delta of Vietnam, two in Thailand and Madagascar, and three in the Vietnam highlands (Table 3). Higher AIV prevalence was associated with a shorter distance to a water body in the Vietnam delta and highlands, as well as in Madagascar. Duck density was found positively associated with AIV in the Vietnam highlands and lower-Northern Thailand. A positive association was found between landscape with predominant rice production and AIV infection in Thailand and Madagascar. Finally, AIV prevalence decreased with increasing distance to the closest major road in the Vietnam highlands.

**Table 1:** Descriptive statistics for the variables analyzed in the four study sites.

	Lower-Northern Thailand	Red River Delta, Vietnam	Vietnam highlands	Lake Alaotra, Madagascar
	Median (IQR^a^)	Median (IQR)	Median (IQR)	Median (IQR)
Chicken density (birds/km^2^)	261.2 (121.4–570.7)	2836.0 (1983.0–3532.0)	146.9 (79.7–239.6)	10.9 (10.3–46.6)
Duck density (birds/km^2^)	4.2 (0.1–40.5)	663.8 (497.2–978.7)	29.5 (16.2–75.9)	9.2 (4.6–11.8)
Percentage of rice fields	0.5 (0.2–0.7)	1 (0.5–1)	0.2 (0–0.4)	0.4 (0.2–0.8)
Water distance (m)	265.9 (113.7–690.1)	1691.0 (811.7–2671.0)	8948.0 (4098.0–15500.0)	740.2 (250.6–2195.0)
Road distance (m)	3300.0 (852.5–6724.0)	3728.0 (1533.0–5909.0)	7897.0 (2838.0–16600.0)	594.5 (377.3–3385.0)
Human population density (persons/km^2^)	100.4 (70.3–116.0)	799.8 (573.6–975.1)	83.9 (55.6–110.4)	38.2 (36.9–53.3)

a interquartile range.

**Table 2:** Results of the spatial univariate logistic models for variables associated with H5N1 confirmed outbreaks in Thailand, H5 seroprevalence in Vietnam and low pathogenic AIV seroprevalence in Madagascar.

	Lower-Northern Thailand	Red River Delta Vietnam	Vietnam highlands	Lake Alaotra, Madagascar
Chicken density^a^ (birds/km^2^)	0.500^b^ (0.152)^c^	0.902 (1.012)	2.675 (0.985)	0.840 (0.364)
	*p* d = 0.001	*p* = 0.376	*p* = 0.007	*p* = 0.022
Duck density^a^ (birds/km^2^)	0.362 (0.070)	0.717 (0.922)	2.439 (0.615)	0.819 (0.523)
	*p*<0.001	*p = *0.439	*p*<0.001	*p* = 0.119
Rice predominant	1.103 (0.339)	–0.303 (0.487)	0.466 (0.493)	0.453 (0.261)
	*p* = 0.001	*p* = 0.535	*p* = 0.346	*p* = 0.085
Water distance^a^ (m)	–0.227 (0.142)	–1.010 (0.457)	–0.983 (0.427)	–0.192 (0.110)
	*p* = 0.110	*p* = 0.030	*p* = 0.023	*p* = 0.082
Road distance^a^ (m)	–0.151 (0.131)	–0.026 (0.390)	–0.349 (0.283)	–0.309 (0.202)
	*p* = 0.251	*p* = 0.948	*p* = 0.219	*p* = 0.128
Human population density^a^ (persons/km^2^)	0.336 (0.147)	–0.016 (1.123)	0.330 (0.301)	0.096 (0.551)
	*p* = 0.023	*p* = 0.989	*p* = 0.275	*p* = 0.861

a log-transformed variables.

b coefficient estimated from the spatial logistic regression model.

c standard-error of the coefficient estimated.

d *p*-value of the Wald test.

**Table 3:** Results of the spatial multivariate logistic models for variables associated with H5N1 confirmed outbreaks in Thailand, H5 seroprevalence in Vietnam and low pathogenic AIV seroprevalence in Madagascar.

	Lower-Northern Thailand	Red River Delta Vietnam	Vietnam highlands	Lake Alaotra, Madagascar
Duck density^a^ (birds/km^2^)	0.308^b^ (0.074)^c^		2.541 (0.509)	
	*p* d<0.001		*p*<0.001	
Rice predominant	0.749 (0.350)			0.926 (0.294)
	*p* = 0.033			*p* = 0.002
Water distance^a^ (m)		–1.010 (0.457)	–1.327 (0.412)	–0.380 (0.124)
		*p* = 0.030	*p* = 0.002	*p* = 0.003
Road distance^a^ (m)			–0.530 (0.238)	
			*p* = 0.028	
Human population density^a^ (persons/km^2^)				
*p χ^2^* e	0.279	0.997	0.577	0.307

a log-transformed variables.

b coefficient estimated from the spatial logistic regression model.

c standard-error of the coefficient estimated.

d *p*-value of the Wald test.

e *p*-value of Hosmer-Lemeshow Chi-squared test.

The Hosmer–Lemeshow goodness-of-fit tests indicated that all final models fitted the data.

## Discussion

The design of this study enabled a large sample of avian influenza data coming from various agro-environmental settings to be analyzed in parallel. All of the study sites had similar agro-ecosystems featuring the farming of domestic ducks and rice cultivation. The four study sites corresponded to different AIV situations. During the data collection period, HPAI H5N1 virus was circulating widely in lower-Northern Thailand [22] as well as in the Red River Delta of Vietnam [32], while only sporadic outbreaks occurred in the Vietnam highlands [23]. In Lake Alaotra (Madagascar highlands), no HPAI outbreaks have been reported but low pathogenic AIV are known to circulate [27]. The present study is, to our knowledge, the first to use the same analytical and statistical approach to examine the distribution of risk factors of avian influenza in four study sites located in three different countries. This made it possible to compare the influence of the same set of agro-environmental determinants on the circulation of avian influenza viruses in different contexts (Table S1).

On the one hand, this multisite study shows that there is a common pattern among the study sites regarding the role of local agro-environmental characteristics in AIV circulation. Indeed, despite the heterogeneity of settings and case definitions (laboratory-confirmed HPAI H5N1 outbreaks in Thailand, H5 seropositivity in Vietnam, and low pathogenic AIV seropositivity in Madagascar), the statistical significance and the sign of the associations were constant across study sites for all tested factors in the univariate analysis (Table 2) with the exception of the Red River Delta (Vietnam). The presence of a common pattern across study sites is also reinforced by the results of the multivariate analysis, which confirmed the importance of three previously identified drivers [6], namely proximity to water bodies, predominance of rice paddy fields and duck density.

On the other hand, the present study helps to refine current understanding of avian influenza by identifying site-specific combinations of agro-environmental factors. These results can be useful for better tailoring surveillance strategies to local conditions. Indeed, we found that the general pattern of risk factors which has been so far evidenced at a broad scale corresponds at a local scale to specific combinations which may differ between study sites.

In lower-Northern Thailand, the multivariate model evidenced that duck density and rice paddy fields were important drivers of HPAI H5N1 outbreaks at the village level. This finding is consistent with a previous study carried out using a coarser epidemiological unit (sub-district) in Thailand [21]. However, it contrasts with the results of a recent study by Van Boeckel et al. [13] showing that the presence of water from rivers and flooding tended to replace rice cropping intensity as a driver of AIV circulation at a fine spatial resolution (village). The authors of this study suggest that the association between rice cultivation and HPAI H5N1 outbreaks observed in Thailand actually may be mainly explained by the frequent flooding of rice fields in intensive systems, which in turn contributes to HPAI H5N1 spread through waterborne transmission [13]. The discrepancy between our results and the Van Boeckel et al. study may derive from a difference in the explanatory variable, i.e. distance to water bodies *vs.* proportion of area covered by lakes, rivers or floods in a 1 km neighborhood around each village location. In addition, the variable we used did not capture flooded areas which coincided with HPAI H5N1 outbreaks and which were specifically targeted by Van Boeckel et al. [13], [39].

In the Red River Delta of Vietnam, we found that higher H5 prevalence at the village level was associated with a shorter distance to water. This is worth noting as none of the studies carried out so far in Vietnam have found this association significant [12], [16], [19]. In the Red River Delta, water bodies may be contaminated by AIV through the feces of ducks which range near them or through infected dead birds thrown into the water by farmers [16]. Contaminated drinking water was proved to be an efficient route by which AIV can be transmitted to poultry [40]. We did not evidence any other predictors in this study site. This may be partly explained by the impact of massive vaccination campaigns which have been implemented from the end of 2005 in the Red River Delta [32]. Vaccination campaigns may have disturbed the overall spatial pattern of H5 circulation in poultry, rendering its analysis very challenging. Although only non-vaccinated poultry were selected for our analysis, they benefited from “herd immunity”, and were thus partially protected against infection. This may be the reason why we did not find an association between poultry densities and AIV circulation. We also did not find any association between predominance of rice cultivation and H5 circulation. Rice is cultivated throughout the Red River Delta, and data showed little variability in the percentage of rice fields present around villages (Table 1). While rice cultivation alone did not appear to be a significant variable in our study, local variations in the number of rice cycles practiced per year may be influencing the spatial pattern of H5 avian influenza circulation in the study area [18]. As we intended this study to be a comparative work, we decided not to test the effect of rice cropping intensity because this would not have been meaningful for the Vietnam highlands and Madagascar, where only one rice cycle is produced each year. This question nonetheless should be investigated further. It could be integrated into much needed studies of the spatial pattern of H5 circulation in the presence of vaccination in Vietnam.

In contrast with the Red River Delta, we identified an effect of duck density on H5 circulation in the Vietnam highlands. Combined with observations from lower-Northern Thailand, this finding confirms that in the absence of poultry vaccination, domestic ducks play a key role in the circulation of H5 viruses. As in the Red River Delta, we also found in the Vietnam highlands an increased H5 seroprevalence at shorter distance to water bodies. In addition, distance to major roads was found to be a key variable, and one which was unique to the situation of the Vietnam highlands. In this site, which is characterized by sparse habitations and traditional poultry farming systems, the national road which connects the study area (Ha Giang province) to China may have favored H5 virus introduction from Yunnan. HPAI H5N1 is indeed considered endemic in China despite vaccination programs. The illegal import of live poultry from China is important, and veterinary controls at the border have limited impact on this trade. The risk of direct or indirect exposure of the Vietnamese poultry population to the HPAI H5N1 virus released by infected poultry illegally imported from China has been assessed as high [41].

It is striking that in Lake Alaotra, Madagascar, several risk factors found in Southeast Asia also are present and play a significant role in the circulation of low pathogenic avian influenza viruses. Indeed, we found that variations of low pathogenic AIV seroprevalence in Lake Alaotra were largely correlated with distance to wetlands and predominance of rice fields around poultry farms. Experimental studies have proven that low pathogenic AIV can remain infective in water for longer periods of time than HPAI H5N1 viruses [14]. It is probable that water from the lake, rivers and rice paddy fields play an important role in the transmission dynamics of low pathogenic AIV in the poultry population of Lake Alaotra through an oral-fecal route. In Lake Alaotra, AIV circulation also may be favored on farms located close to water bodies by the presence in these areas of important wild waterfowls congregations [36], which are a natural reservoir of low pathogenic AIV [42]. Another interesting characteristic of Lake Alaotra is the presence of flocks of ducks and geese, which are brought during the day to graze on rice paddy fields like in Southeast Asia. We thus expected to observe an association between duck density and AIV seroprevalence in Madagascar similar to that found in the Southeast Asian sites in relation to H5 subtype. However, we did not find this association. This may be due to an insufficient resolution of the duck density variable, which was collected at the commune level and varied little between farms in Madagascar. Another possible explanation is that the strength of the association of duck density with low pathogenic AIV is lower than that with HPAI H5N1 and the statistical power of our study did not allow its detection. An alternative interpretation of our results is that, as suggested by a recent study on HPAI H5N1 in Thailand [13], duck density may be associated with the risk of AIV only when ducks are raised in intensive systems, or when their numbers are large for extensive systems. In Madagascar, flock size is considerably lower than in Vietnam or Thailand (10 ducks on average in Madagascar *vs* 1000 to 4000 in Thailand and Vietnam), and the low duck density (median: 9.2 ducks/km^2^, interquartile range IQR: 4.6–11.8) may not reach the threshold that would be necessary to influence AIV seroprevalence in poultry populations [43]. In contrast, in the Vietnam highlands, where duck farming also is mainly extensive but with higher densities (median: 29.5 ducks/km^2^, IQR: 16.2–75.9), we found a positive association between duck density and H5 seroprevalence. In lower-Northern Thailand, although the median value (4.2 ducks/km^2^) was the lowest among the study sites, duck density had a wide range (IQR: 0.1–40.5) and included both extensive and large-scale intensive systems. Here also we found an effect of duck density on the probability of an HPAI H5N1 outbreak. Our observations from three contrasting agro-ecosystems thus support the hypothesis that for extensive systems, duck density influences AIV circulation only when it is above a threshold which may allow frequent contacts between ducks and between ducks and chickens. Massive vaccination campaigns carried out in the Red River Delta, Vietnam, impede from generalizing this hypothesis to this study site and thus to intensive duck farming systems.

It is noteworthy that we did not find avian influenza to be associated with human population density in any of the four study sites. This contrasts with previous studies carried out at the village level in Indonesia [8] and Thailand [13]. We considered two possible explanations. Firstly, the association that has been observed between the risk of HPAI H5N1 outbreaks and human population density may be a reflection of the increased detection sensitivity of surveillance in highly populated areas. This might be particularly applicable to studies based on avian influenza data collected through passive surveillance systems. It is reasonable to assume outbreaks are more likely to be detected and reported in more densely populated places. The fact that we did not observe an association between AIV circulation and human population density may simply confirm that such detection bias was limited in our study, which had a much more limited spatial extent. This hypothesis sounds reasonable in Vietnam and Madagascar, as AIV data were collected using cross-sectional serological surveys designed without any restriction on village size or accessibility. This hypothesis also makes sense in Thailand, where H5N1 outbreaks were detected by comprehensive active detection surveys involving several hundred thousand volunteers searching door-to-door for evidence of infection in even the smallest villages and remote areas. Secondly, human population density has also been used as a surrogate for poultry markets and areas of intensive poultry trading activities that may in turn support an increased risk of AIV transmission through flows of contaminated poultry or fomites [21]. From our study, one also could hypothesize that human population density may not be a relevant proxy for poultry trading activities in fine-scale studies focusing on a limited geographic area. When working with fine resolution data (farm or village level) on small study areas, it may be necessary to use instead variables which reflect more closely the organization of poultry trade on the ground. The distance to the closest wet markets or the presence of a poultry trader in the village [16] could constitute better risk indicators for avian influenza than human population density in this case.

This study had some limitations. Firstly, each study site presented specific constraints. In Vietnam and Madagascar, the presence of antibodies against AIV was evaluated using commercial Elisa tests with imperfect performance; this may have resulted in the overestimation of seroprevalences [24]. In Madagascar, the poultry density data may have had an insufficient resolution (commune) given the scale of analysis (farm). In addition, the study protocol in the Red River Delta (Vietnam) included 83 villages, resulting in a limited statistical power. This may have led us to erroneously conclude that some potential risk factors did not have effect on avian influenza prevalence in this site when they did. Secondly, not all data were collected according to the same procedures across the four study sites. This limited the possibility of a pooled analysis. In lower-Northern Thailand and Vietnam highlands, we believe that the data collection process allowed to obtain a fair picture of AIV circulation over a one year period. Indeed, it is acknowledged [44] that HPAI H5N1 surveillance in Thailand was comprehensive during the study period we considered (second wave of epidemics). In Vietnam highlands, despite time variations in their numbers, poultry samples were taken monthly over more than one year and provided an acceptable overall view. In the two other study sites, however, we captured only a partial picture of the AIV situation with poultry sera collected in August and May only in Madagascar, and from December to July in the Red River Delta (Vietnam). A seasonal pattern has been observed for occurrence of HPAI H5N1, with higher risk of HPAI H5N1 outbreaks from November to January and April to June [45]. Temporal variations were also previously described for seroprevalence of low pathogenic avian influenza, including H9N2 [46] and cannot be excluded in Madagascar highlands [36]. Given this discrepancy in data collection across study sites, we may thus have compared the yearly situation captured in Thailand and Vietnam highlands, with a “high-risk” period in the Red River Delta (Vietnam) and with an AIV situation corresponding to the dry season only in Madagascar. The temporal pattern of AIV circulation in poultry could be explained by different factors including varying contacts between poultry and wild birds, virus survival under climatic conditions, proportion of land covered by flooding [39], but also seasonality of poultry production and trade [36]. Despite the fact that data on the distribution in space and time of both AIV and all these factors are very difficult to obtain at high resolution, further studies should seek to include simultaneously agro-environmental, climatic and poultry trade related variables to eliminate any possible confounding. The difference in the size of the study areas also made it difficult to compare the range of spatial autocorrelation for avian influenza between sites. However, it is noteworthy that the extent of the spatial correlation we found in Thailand (35 km) and Vietnam (10 and 25 km) is consistent with results from previous studies carried out in the same countries [30], [47]. The AIV data were nevertheless obtained from good-quality protocols which guaranteed the representativeness of the respective poultry populations in each study site [23], [27], [32]. Great care was also undertaken to obtain the highest quality ecological data in an effort to offset potential uncertainties associated with multi-country ecological comparisons.

Despite these limitations, to our knowledge this analysis is the first to compare risk factors for avian influenza across a variety of countries using fine-scale field data. We found a great deal of consistency across much of the study sites. Results confirmed the importance of wetlands-rice-ducks agro-ecosystems in the epidemiology of H5 avian influenza in Southeast Asia, and also showed that a similar agro-ecosystem contributed to the circulation of low pathogenic AIV in Madagascar. Mapping these agro-ecosystems could be useful to identify hot spots of AIV circulation across various countries. Moreover, this multisite study revealed that the relative contribution of these risk factors in the circulation of AIV differed between local environmental conditions. The analysis of agro-environmental variables collected at a very fine scale may allow the identification of villages and farms presenting a high risk for AIV circulation, and thus could help to tailor surveillance and control measures to local conditions.

## Supporting Information

Figure S1 **Temporal distribution of HPAI H5N1 confirmed outbreaks in lower-Northern Thailand (A), and of percentages of poultry samples taken in the Red River Delta (B) and Vietnam highlands (C).**
(PDF)

Figure S2 **Spatial correlogram of H5N1 presence or absence in lower-Northern Thailand (A), H5 seroprevalence in the Red River Delta, Vietnam (B), H5 seroprevalence in the Vietnam highlands (C) and low pathogenic AIV seroprevalence in Lake Alaotra, Madagascar highlands (D).**
(TIF)

Figure S3 **Omnidirectional variograms computed using the standardized residuals derived from the spatial generalized linear model used in lower-Northern Thailand (A), in the Red River Delta, Vietnam (B), in the Vietnam highlands (C) and in Lake Alaotra, Madagascar highlands (D).** The dashed lines show the pointwise 95% limits constructed from the Monte Carlo 999 simulations; circles represent the empirical variogram.
(TIF)

Table S1 **Summary statistics for the variables examined in the four study sites: lower-Northern Thailand (A), the Red River Delta, Vietnam (B), the Vietnam highlands (C), and Lake Alaotra, Madagascar highlands (D).**
(DOCX)

Material S1 **Description of the spatial generalized linear model used to study the effect of agro-environmental determinants on avian influenza circulation.**
(PDF)
