# Supplementary material for: Agro-Environmental Determinants of Avian Influenza Circulation: A Multisite Study in Thailand, Vietnam and Madagascar
Source: PLoS One. 2014 Jul 16;9(7):e101958. doi: 10.1371/journal.pone.0101958 (PMC4100877; doi:10.1371/journal.pone.0101958)

Figure S1. Temporal distribution of HPAI H5N1 confirmed outbreaks in lower-Northern Thailand (A), and of percentages (%) of poultry samples taken in the Red River Delta (B) and Vietnam highlands (C).

(A) lower-Northern Thailand (n=1032 villages monitored)

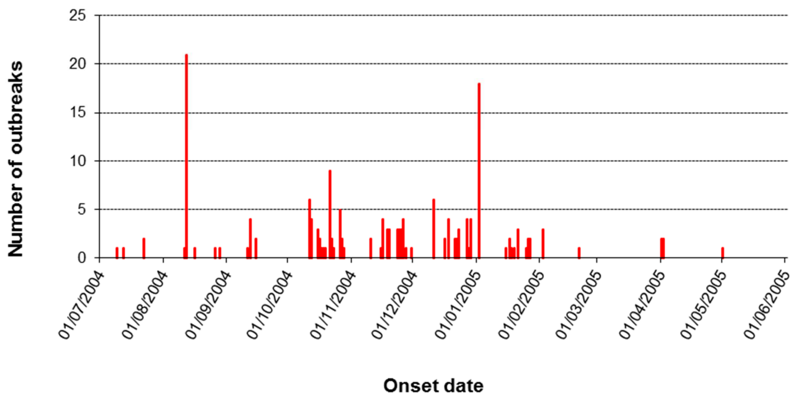

(B) Red River Delta, Vietnam (n=3234 samples)

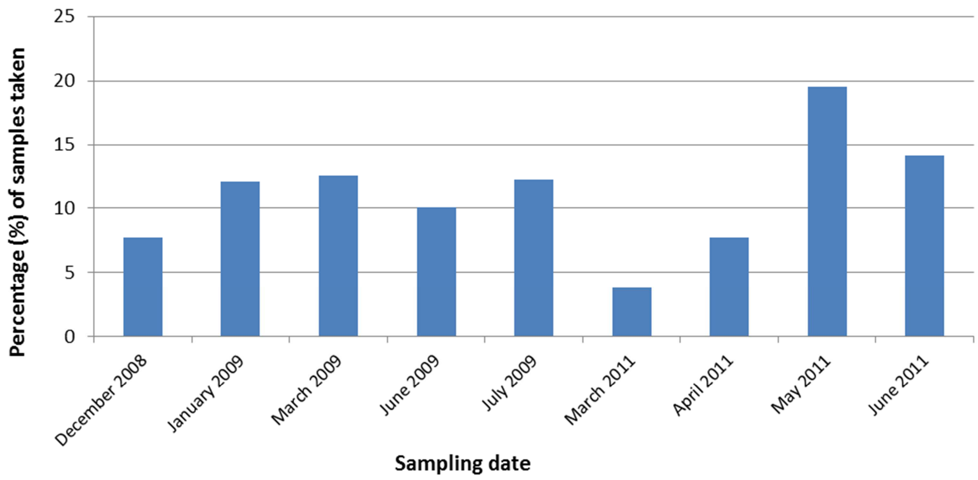

(C) Vietnam highlands (n=1531 samples)

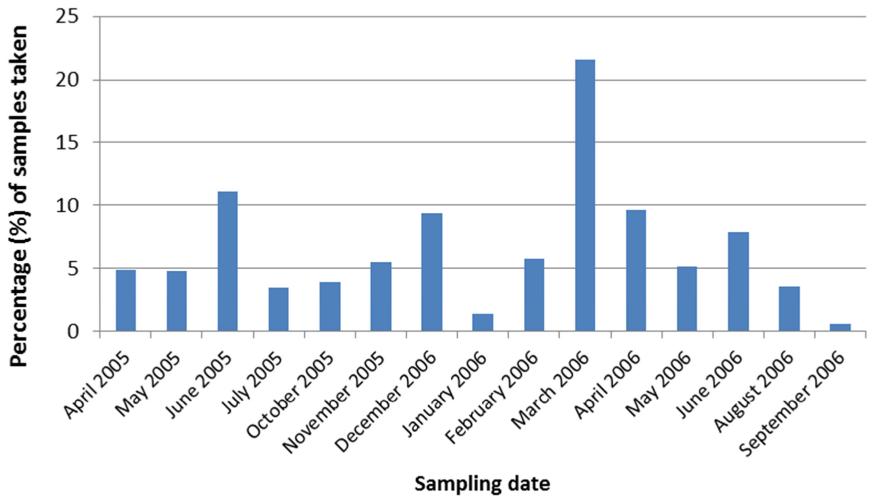

Supplement: Figure S1 — Temporal distribution of HPAI H5N1 confirmed outbreaks in lower-Northern Thailand (A), and of percentages of poultry samples taken in the Red River Delta (B) and Vietnam highlands (C). (PDF) [file pone.0101958.s001.pdf]
