# Supplementary figures and images for: Agro-Environmental Determinants of Avian Influenza Circulation: A Multisite Study in Thailand, Vietnam and Madagascar
Source: PLoS One. 2014 Jul 16;9(7):e101958. doi: 10.1371/journal.pone.0101958 (PMC4100877; doi:10.1371/journal.pone.0101958)

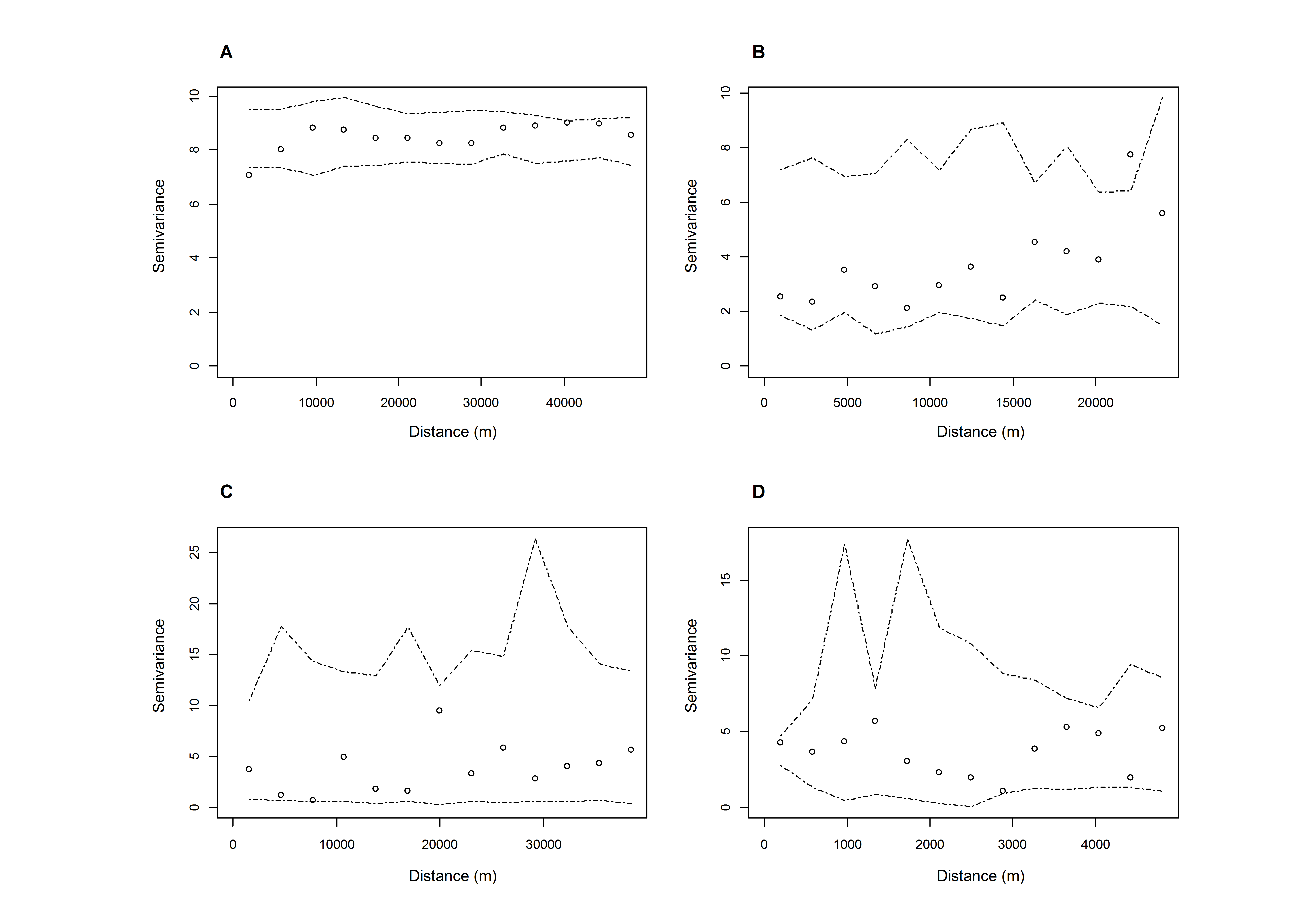

Supplement: Figure S3 — Omnidirectional variograms computed using the standardized residuals derived from the spatial generalized linear model used in lower-Northern Thailand (A), in the Red River Delta, Vietnam (B), in the Vietnam highlands (C) and in Lake Alaotra, Madagascar highlands (D). The dashed lines show the pointwise 95% limits constructed from the Monte Carlo 999 simulations; circles represent the empirical variogram. (TIF) [file pone.0101958.s003.tif]
