# Supplementary material for: Agro-Environmental Determinants of Avian Influenza Circulation: A Multisite Study in Thailand, Vietnam and Madagascar
Source: PLoS One. 2014 Jul 16;9(7):e101958. doi: 10.1371/journal.pone.0101958 (PMC4100877; doi:10.1371/journal.pone.0101958)
Supplement: Table S1 — Summary statistics for the variables examined in the four study sites: lower-Northern Thailand (A), the Red River Delta, Vietnam (B), the Vietnam highlands (C), and Lake Alaotra, Madagascar highlands (D). (DOCX) [file pone.0101958.s004.docx]

**Table S1. Summary statistics for the variable examined in the four study sites : lower-north Thailand (A), the Red River Delta, Vietnam (B), the Vietnam highlands (C), and Lake Alaotra, Madagascar highlands (D).**

(A) Lower-north Thailand

|  | Median for positive villages (IQR) (n=163) | Median for negative villages (IQR) (n=869) |
| --- | --- | --- |
| Chicken density (birds/km²) | 353.2 (175.7 – 713.6) | 243.1 (111.9 – 557.6) |
| Duck density (birds/km²) | 16.4 (2.3 – 240.7) | 2.8 (0.0 – 28.1) |
| Proportion of land occupied by rice fields around villages | 0.6 (0.4 – 0.8) | 0.5 (0.2 – 0.7) |
| Water distance (m) | 195.6 (90.9 – 663.4) | 271.9 (119.5 – 706.4) |
| Road distance (m) | 3118.0 (855.7 – 6058.0) | 3362.0 (852.4 – 6994.0) |
| Human population density (persons/km²) | 105.9 (81.5 – 116.0) | 98.7 (68.7 – 116.0) |

(B) Red River Delta, Vietnam

|  | Median for positive villages (IQR) (n=47) | Median for negative villages (IQR) (n=36) |
| --- | --- | --- |
| Chicken density (birds/km²) | 2379.0 (1983.0 – 34636.0) | 2449.0 (1994.0 – 3584.0) |
| Duck density (birds/km²) | 693.6 (489.7 – 988.4) | 644.2 (534.0 – 894.4) |
| Proportion of land occupied by rice fields around villages | 1.0 (0.5 – 1.0) | 1.0 (0.6 – 1.0) |
| Water distance (m) | 1612.0 (768.4 – 2343.0) | 2000.0 (1113.0 – 2817.0) |
| Road distance (m) | 3796.0 (1569.0 – 5608.0) | 3611.0 (1513.0 – 6104.0) |
| Human population density (persons/km²) | 852.6 (588.0 – 1031.0) | 777.3 (567.2 – 871.8) |

(C) Vietnam highlands

|  | Median for positive villages (IQR) (n=20) | Median for negative villages (IQR) (n=147) |
| --- | --- | --- |
| Chicken density (birds/km²) | 285.0 (206.3 – 316.2) | 133.3 (78.5 – 223.4) |
| Duck density (birds/km²) | 81.6 (44.8 – 103.9) | 27.9 (14.5 – 53.9) |
| Proportion of land occupied by rice fields around villages | 0.2 (0.00 – 0.4) | 0.1 (0.0 – 0.3) |
| Water distance (m) | 2425.0 (953.0 – 9248.0) | 9881.0 (5157.0 – 16120.0) |
| Road distance (m) | 1445.0 (389.4-3377.0) | 9704.0 (4180.0 - 18340.0) |
| Human population density (persons/km²) | 181.4 (46.3 – 268.7) | 80.6 (52.7 – 105.0) |

(D) Lake Alaotra, Madagascar highlands

|  | Median for positive farms (IQR) (n=74) | Median for negative farms (IQR) (n=73) |
| --- | --- | --- |
| Chicken density (birds/km²) | 23.7 (10.3 – 46.6) | 10.9 (10.3 – 36.4) |
| Duck density (birds/km²) | 11.8 (4.6 – 11.8) | 9.2 (1.7 – 11.8) |
| Proportion of land occupied by rice fields around villages | 0.4 (0.2 – 1.0) | 0.4 (0.1 – 0.6) |
| Water distance (m) | 756.2 (124.2 – 2181.0) | 740.2 (340.6 – 2211.0) |
| Road distance (m) | 632.9 (389.4 – 3421.0) | 579.1 (370.2 – 1080.0) |
| Human population density (persons/km²) | 38.2 (36.9 – 58.7) | 38.3 (37.0 – 41.8) |
