# Supplementary material for: Agro-Environmental Determinants of Avian Influenza Circulation: A Multisite Study in Thailand, Vietnam and Madagascar
Source: PLoS One. 2014 Jul 16;9(7):e101958. doi: 10.1371/journal.pone.0101958 (PMC4100877; doi:10.1371/journal.pone.0101958)
Supplement: Material S1 — Description of the spatial generalized linear model used to study the effect of agro-environmental determinants on avian influenza circulation. (PDF) [file pone.0101958.s005.pdf]

Supplementary Material S1. Description of the spatial generalized linear model used to study the effect of agro-environmental determinants on avian influenza circulation.

The statistical analysis was based on spatial generalized linear mixed models, which were run using the MASS package of the R software. The spatial dependency between observations was accounted for by introducing a correlation structure in the models.

Formally, if Y_i and Y_j are the observations of the response variable at location i and j, the model can be written as:

$$\begin{cases} \log\left(\frac{E(Y_i)}{1 - E(Y_i)}\right) = x_i\,\beta \\ \mathrm{corr}(Y_i\,, Y_j) = \exp\left(-\frac{d_{ij}}{r}\right) \end{cases}$$

where $E(Y_i) = P(Y_i = 1)$ is the expectation of Y_i, β is the coefficients for fixed effects, x_i is the explanatory variables for the i-th observation, $\mathrm{corr}(Y_i\,, Y_j)$ is the correlation between Y_i and Y_j, d_{ij} is the Euclidian distance between locations i and j, and r is the range of the spatial correlation estimated from the data.
